# Study of the influence of crossbite on the size of the apical area in a child population by analyzing panoramic X-ray

**DOI:** 10.4317/jced.54915

**Published:** 2018-08-01

**Authors:** Nazareth Rodríguez-Peinado, Montserrat Diéguez-Pérez, Gloria Saavedra-Marbán, María-Rosa Mourelle-Martínez

**Affiliations:** 1 DMD, MS, PhD, Clinical Assistant Professor of Pediatric Dentistry, Department of Specialties Dental Clinics, Faculty of Dentistry, Complutense University of Madrid; 2 DMD, MS, PhD, Associate Professor of Pediatric Dentistry, Department of Specialties Dental Clinics, Faculty of Dentistry, Complutense University of Madrid. Assistant Professor in the Dentistry Department. School of Biomedical Science. European University of Madrid; 3 DMD, MS, PhD, Associate Professor of Pediatric Dentistry, Department of Specialties Dental Clinics, Faculty of Dentistry, Complutense University of Madrid; 4 DMD, MS, PhD, Contract Doctor Professor of Pediatric Dentistry, Department of Specialties Dental Clinics, Faculty of Dentistry, Complutense University of Madrid

## Abstract

**Background:**

The apical area is the space in the maxillary bones that contains teeth during formation and is subsequently occupied by the apices of the permanent teeth. Its dimensions are easy to perceive and determine by observing a panoramic X-ray. Our objective was to analyze the influence of crossbite on the size of the anterior and mesial apical area in Caucasian children.

**Material and Methods:**

Based on the ortopantomograph of 353 patients in mixed dentition and crossbite, the sizes of the apical areas of the four hemiarches were studied using the Tps Dig Version 2® computer program. These data were subjected to statistical analysis using the SPSS 22.0 for Windows program and applying the methods of descriptive statistics of quantitative variables, the Kolmogorov-Smirnov test, the non-parametric test Mann-Whitney-Wilcoxon test, and the paired Student t-test.

**Results:**

In the group of boys, average values in the superior-mesial, superior-anterior, inferior-mesial and inferior-anterior apical areas of the crossbite were 173.43, 99.85, 180.32 and 87.56 respectively, with the lower values being in the hemiarch without malocclusion. In the group of girls, for the same apical areas, average values were 165.64, 94.24, 168.62 and 83.34 respectively, with all the highest values being in the hemiarch with crossbite, except for the inferior-mesial apical area. Statistically significant differences were found in the hemiarch with crossbite between both genders in the superior-anterior, inferior-anterior and inferior-mesial apical areas, with the significance being 0.001, 0.029 and 0.001 respectively, while in the hemiarch without malocclusion significance was observed in the superior-mesial, superior-anterior and inferior-mesial apical areas, with values of 0.004, 0.001 and 0.004, respectively.

**Conclusions:**

Crossbite affects the size of the anterior apical area in both arches and in both genders. The mesial apical area is influenced by this malocclusion in the jaw in boys and in the maxilla girls.

** Key words:**Apical area, ortopantomography, crossbite.

## Introduction

The apical area is the space in the maxillary bones that contains teeth during formation. It can be divided into three sub-areas: anterior, mesial and posterior. Their mesio-distal dimension affects their size. Each of these sub-areas has specific functions and characteristics and may be prone to reduction caused by influences such as inter-proximal caries and oral habits. A genetic component may also have a decisive influence on the configuration and total growth of the maxillae (1,2).

It is clear that diagnoses of crossbite in the young child are increasing, as are the alterations that this causes in the stomatognathic system. There have been few studies that relate this malocclusion to the apical area, since they have only been used as a predictor of tooth eruption in situations with compromised space in the arch. Pulido et al. observed that the dimensions of the apical area and its relations with developing permanent teeth are easy to perceive and determine by observing a panoramic X-ray (2). Our research is of interest because the size of the apical areas in patients with crossbite might help determine the influence of chewing on bone development. We consider whether there are quantitative variations in the anterior and mesial apical areas between the hemiarches with and without crossbite.

## Material and Methods

An observational and cross-sectional cohort study was carried out. The total sample consisted of radiographic and photographic records of 752 Caucasian patients who attended a Radiological Center. Selection was random and based on a non-probabilistic sampling of consecutive cases. The sample selection criteria were: patients in mixed dentition, with unilateral crossbite, aged between 6 and 9 years, with photographic and radiographic records of sufficient quality for the diagnosis of malocclusion and observation of the study area, no systemic diseases, no syndromes or congenital bucco-facial malformations, no extensive caries or restored carious pathology, no alterations in tooth development or ectopic eruptions of any of teeth involved in the measurements, no premature dental losses and not receiving corrective treatment for previous or current malocclusion. All of them were required to have an informed consent form signed by their parents or legal guardians.

The principal researcher carried out a photographic and radiographic diagnosis protocol, considering that there was crossbite when the upper vestibular cusp of a tooth occluded lingually from the lower vestibule. Cases with 1 to 6 teeth involved in the crossbite were accepted as valid. All ortopantomographs were examined using the Tps Dig Version 2® software, marking lines corresponding to the demarcations of the anterior and mesial apical areas in both arches.

**Figure 1:**
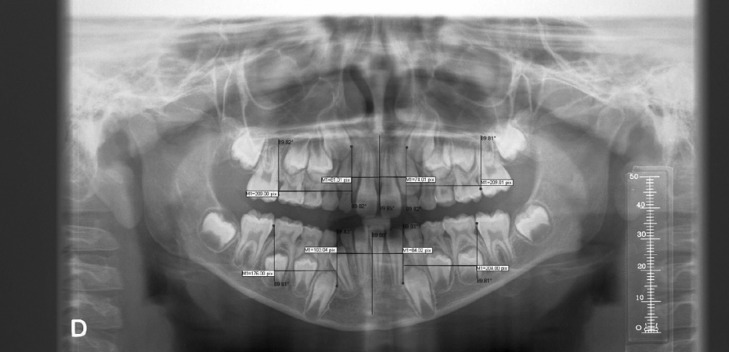
Measurements of the study variables upper mesial, upper anterior, lower mesial and lower anterior apical area.

The study variables measured in pixels were (Fig. 1):

•Mesial upper right/left apical area: distance from the most prominent and mesial point of the vertical line of the first permanent upper right/left molar to the most prominent and mesial point of the vertical line of the permanent upper right/left canine.

•Anterior upper right/left apical area: distance from the most prominent and mesial point of the vertical line of the permanent upper right/left canine to the most prominent and mesial point of the vertical line of the anterior nasal spine.

•Mesial lower right/left apical area: distance from the most prominent and mesial point of the vertical line of the first permanent lower right/left molar to the most prominent and mesial point of the vertical line of the permanent lower right/left canine.

•Anterior lower right/left apical area: distance from the most prominent and mesial point of the vertical line of the permanent lower right/left canine to the most prominent and mesial point of the vertical line of the mandibular symphysis.

The data were collected on an Excel sheet and the statistical analysis was used the SPSS 22.0 program for Windows, applying the following methods: descriptive statistics of quantitative variables for description of the samples (median, standard deviation, maximum, minimum and median values); descriptive statistics obtaining frequencies and category percentages in relation to gender; the Kolmogorov-Smirnov test to determine the distribution of the quantitative variables of the study; the non-parametric Mann-Whitney-Wilcoxon test to compare the measurement of a quantitative variable between two groups; the paired Student t-test. In each of the results, a check was made for significant differences with a confidence interval of 95%. An error estimate was performed 20 days after the last measurement, randomly selecting 20% of the total sample.

## Results

The analysis covered the panoramic X-rays of 353 patients (153 boys and 200 girls) who met the criteria for inclusion. 119 were age 6, 84 were age 7, 88 were age 8, and 62 were age 9. We analyzed the values obtained from the measurements of the eight apical areas in both hemiarches, with and without lateral crossbite, according to the patient’s gender.

In the group of boys, the highest mean value for the upper mesial apical area, 173,43, was found in the hemiarch without crossbite, while the highest mean values in the upper anterior, lower anterior and lower mesial apical areas were found in the hemiarch with crossbite, with values of 99.85, 87.56 and 180.32 respectively. In the group of girls, the highest mean value for the lower mesial apical area, 168.62, was found in the hemiarch without crossbite, while the highest mean values in the upper mesial, upper anterior and lower anterior apical areas were found in the hemiarch with crossbite, with values of 165.64, 94.24 and 83.34 respectively.

When each of the apical areas in the hemiarch with crossbite was compared between boys and girls, no statistically significant differences were found in the upper mesial apical area. But in the upper anterior, lower anterior and lower mesial apical areas, significant differences were found, of 0.001, 0.029 and 0.001 respectively. While in the hemiarch without malocclusion, no statistically significant differences were found in the lower anterior apical area, significant differences between boys and girls were found in the upper mesial, upper anterior and lower mesial apical areas, of 0.004, 0.001 and 0.004 respectively ( Table 1).

**Table 1:**
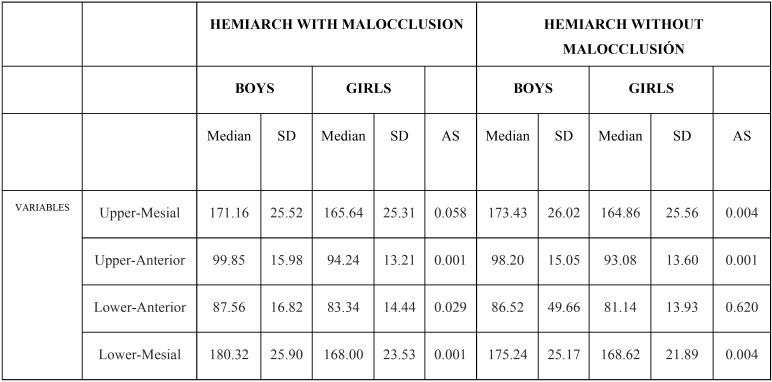
Measurements of the apical areas in the hemiarch with and without malocclusion in both genders. Descriptive statistics of the variables: Median. Standard deviation (SD). Comparison between apical areas in both hermiarches and by gender: Analysis of significance according to the Mann-Whitney-Wilcoxon Test (AS).

## Discussion

There is extensive literature on crossbite but, to our knowledge, no studies have evaluated the anterior and mesial apical areas in children with unilateral crossbite using ortopantomography. Since this examinations is requested routinely, we consider it could provide information regarding compromised space in the arch and could determine if malocclusion is likely to interfere in the bone development of the hemiarches.

The only study we have found in the literature measuring apical areas is Pulido *et al.* (2) who also use panoramic X-rays. However, Pulido *et al.* (2) analyze the relation between the size of the anterior mandibular apical area and antero-inferior crowding, while we study the anterior and mesial apical areas in both arches taking into account the presence of unilateral crossbite rather than antero-inferior crowding. The age range of the sample is very similar in both studies, but Pulido *et al.* (2) included only 35 patients in their research. In this case, a small apical area was the most frequent (57.1%), with a a high incidence of anterior-lower crowding (62.8%) and with primary crowding being the most common type (77.2%). In 72% of the patients who presented crowding, a small apical area was observed. These results confirmed that the size of the mandibular anterior apical area contributes to anterior-lower crowding. In our study, only the anterior apical areas of both jaws are always influenced by the presence of crossbite. This may be because malocclusion in the anterior area leads to crowding and, according to Pulido *et al.* (2), there seems to be a direct and inversely proportional relationship between the size of the anterior mandible apical area and crowding in the anterior mandible. Therefore, the smaller the size of the apical area, the greater the possibility of crowding.

The disadvantage of panoramic X-ray analysis according to Kambylafkas *et al.* (3) and García-Figueroa *et al.* (4) is the poor quality of the image resulting from distortion, which magnifies the bone and dental structures. Following Pulido *et al.* (2) and McKee *et al.* (5), the dimensions of the apical area and its relations with developing permanent teeth are easy to perceive and determine from a digital panoramic X-ray. We agree with Catié *et al.* (6-7) that it is essential to use the same radiographic equipment to perform all the X-rays in the study. In our study, we used the Orthophos (Siemens) orthopantomographer since, like Schulze *et al.* (8), we consider that it allows reliable reproduction of the measurements of the study variables.

All the radiographs were performed by the same technician trained in the procedure to avoid postural errors since, according to García-Figueroa *et al.* (4), Stramotas *et al.* (9), McKee *et al.* (10) and Larheim and Svanaes (11), inappropriate cephalic position during performance of the technique may result in blurred and distorted images.

Like Schulze *et al.* (12), we consider that the marking of vertical measurement lines from the reference points is reliable and reproducible, as shown by the intra-examiner concordance. However, other authors such as Stramotas *et al.* (9), Larheim and Svanaes (11), Tronje *et al.* (13,14) Habets *et al.* (15) and Laster *et al.* (16) question this reliability.

The measurements in our study are precise because they do not cross the mandibular midline. This is consistent with Catié *et al.* (6,7), who affirm that the distances that cross the middle line have a magnification factor of 1.45-1.85.

## Conclusions

Crossbite affects the size of the anterior apical area in both arches and in both genders. The mesial apical area is influenced by malocclusion in the jaw in boys and in the maxilla in girls. It would be useful to continue studying bone development in patients with unilateral crossbite from the viewpoint of the size of apical areas to determine the influence of mastication on bone growth.
